# Age related changes seen in human cornea in formalin fixed sections and on biomicroscopy in living subjects: A comparison

**DOI:** 10.1002/ca.23488

**Published:** 2019-10-21

**Authors:** Samanta Taurone, Selenia Miglietta, Marialuisa Spoletini, Janos Feher, Marco Artico, Veronica Papa, Roberto Matassa, Giuseppe Familiari, Pietro Gobbi, Alessandra Micera

**Affiliations:** ^1^ IRCCS—Fondazione Bietti Rome Italy; ^2^ Department of Anatomical, Histological, Forensic Medicine and Orthopedics Sciences “Sapienza” University of Rome Rome Italy; ^3^ Department of Sensory Organs “Sapienza” University of Rome Rome Italy; ^4^ Division of Ophthalmology University of Pecs Pecs Hungary; ^5^ Department of Motor Sciences and Wellness University of Naples “Partenope” Naples Italy; ^6^ Department of Biomolecular Sciences University of Urbino “Carlo Bo” Urbino Italy

**Keywords:** age‐related changes, cornea, light microscope, nerve fibers, TEM, VP‐SEM, ICVM

## Abstract

The purpose of our experimental research was to assess the effects of aging on the main corneal structures in healthy corneas. Small, human cornea samples were collected from 20 Caucasian subjects during surgery for traumatic lesions to the eye. Ten subjects were adults (mean age 28 years) and 10 were elderly (mean age 76 years). Morphological analysis was carried out using light microscopy and electron microscopy. Another 40 patients (20 young: mean age < 30 years; 20 elderly: mean age > 70 years) were studied in vivo by confocal microscopy. The resulting images were analyzed qualitatively, quantitatively, and statistically. The basic light microscope revealed a decrease in endothelial cell density with age accompanied by an increase in endothelial cell size. Transmission electron microscopy revealed a corneal thinning and a decrease in the number of corneal stromal cells. A marked decrease in stromal nerve fibers was observed in the older subjects compared to the younger ones. Variable pressure scanning electron microscopy (VP‐SEM) was used to make surface morphological observations and to determine the chemical composition of in vivo hydrated human corneas. Our results showed the effects of aging on normal corneal morphology highlighting the structural diversity of the corneal layers and revealing an age‐related reduction in nerve fibers, thus explaining the decreased corneal sensitivity that may be observed in the elderly. Clin. Anat. 33:245–256, 2020. © 2019 Wiley Periodicals, Inc.

## INTRODUCTION

The corneal compartment is one of the most highly innervated areas of the human body and this specific innervation is supplied by the ophthalmic and the maxillary branch of the trigeminus. The abundant bundles of corneal nerves react to sensitive stimuli and pain, thus playing an important role in the corneal reaction to external environmental conditions. Corneal nerves activate the blinking reflexes that contribute to eye hydration as well as the secretion of neuropeptides, which produce mitogenic effects on the adjacent epithelial cells (Stepp et al., 2017). The nerve endings are a part of the nerve fiber responsible for transducing stimuli into neural signals; therefore, corneal nerve density is directly proportional to corneal sensitivity. Corneal sensitivity changes with age in humans, and a more pronounced decrease can be observed in subjects over the age of 50. The architecture of the corneal nerve fibers has been studied extensively by both light microscopy and electron microscopy (Al‐Aqaba et al., 2010; Stachs et al., 2010). Such studies show that the path is followed by nerve bundles that are distributed radially in the peripheral cornea (He et al., 2010; Marfurt et al., 2010). These nerve bundles, at the level of the limbus, lose their myelin sheath and then extend toward the front center covered only by a thin sheath composed of Schwann cells. Below Bowman's membrane, these nerve bundles form the sub‐epithelial plexus, except when the direction crosses Bowman's membrane at a right angle (Lian et al., 2013). Therefore, these nerve fibers run parallel to the corneal surface, giving rise to a sub‐basal nerve plexus, and branching nerve fibers that reach the corneal epithelium in the form of free nerve endings (Takahashi et al., 2012; Yadav et al., 2012). In the anterior segment of the cornea, innervation density is reduced or absent at both the stromal and endothelial levels (Jonsson et al., 2006; Marfurt et al., 2010). Human corneal fibers undergo rapid degeneration only a few hours after death, meaning that the studies performed to date have significant limitations. A new approach has been introduced to overcome this problem, namely confocal microscopy (CM) procedures, which make it possible to evaluate the corneal innervation in vivo in a noninvasive manner. Given the location of the nerve plexus, CM allows us to obtain images of this layer that may be used to carry out qualitative and quantitative studies of the nerve fibers that are found within it (Tavakoli et al., 2015). However, not all the corneal nerve fibers that radiate from the nerve plexus may be identified using CM (Patel et al., 2009). The innervation of the cornea is fundamental for the maintenance of corneal morphology and function and provides protective mechanisms against factors that might be potentially injurious to the cornea. Denervation and reduced corneal sensitivity are accompanied by impairment of epithelial and endothelial cell function, increased epithelial and endothelial permeability, diminished cell migration, and cell mitosis. Moreover, denervated corneas are prone to epithelial or stromal abnormalities, recurring erosion, impaired wound healing, and infection. Recently, a new layer of the cornea has been identified. It is defined as Dua's layer (DL) and consists of a layer of collagen tissue, impervious to air and almost acellular, just anterior to Descemet's membrane (DM) (Dua et al., 2013). Due to its particular characteristics, this layer was deemed to be different from the deep corneal stroma and was termed the pre‐Descemet's layer (Dua et al., 2013). DL was characterized by Dua et al. and was shown to be strong (Zaki et al., 2015), demonstrated by its ability to resist a pressure of around 500 mm of mercury (Dua et al., 2013, 2014). The layer is made of five to eight compact thin lamellae of Type 1 collagen, has an abundance of Type 6 (long spacing) collagen, is devoid or sparsely populated with keratocytes, is impervious to air, is populated by trabecular cells at the peripheral 350 μm, and continues as the collagen core of the trabecular meshwork (Dua et al., 2015a, 2015b; Dua and Said, 2016). The cornea is characterized by important changes with normal aging. Indeed, numerous age‐related corneal changes have been reported; however, few of these changes are well understood from a clinical standpoint. A distinction must be drawn between physiological conditions (within the “normal limits” of aging) and true pathological conditions, usually affecting the corneas of elderly patients and becomes more susceptible to infection because of a diminished ability to resist various factors. Moreover, certain extracellular matrix molecules act as spatial barriers against nerve fiber extension in the peripheral nervous system. These components include glycosaminoglycans, keratin and chondroitin sulfate, as well as molecules binding lectin and peanut agglutinin. Sometimes it is difficult to distinguish age‐specific alterations from degenerative modifications due to environmental and genetic factors. A considerable number of studies have attempted to demonstrate the changes occurring in corneal innervations (with particular regard to the density of nerves related to age (Dehghani et al., 2014; Gatzioufas et al., 2014). Corneal images acquired by in vivo specular and CM provide clinical information on the health of the cornea endothelium. Indeed, the normal hexagonal shape of endothelial cells is usually affected by age and pathologies. Regarding the corneal stroma, keratocytes play an important role in structural protein remodeling and corneal wound healing (Midena et al., 2007). In the stroma, keratocyte nuclei appear as hyperreflective objects scattered against a dark background. Keratocyte density decreases in each stromal layer with aging. Niederer et al. (2003) reported a 0.9% yearly reduction in anterior keratocyte density and a 0.3% yearly reduction in posterior keratocyte density. During aging, corneal endothelial cell density (ECD) decreases. Corneal endothelial cells play an important role in the maintenance of corneal transparency, in the age‐related decrease in keratocyte density (5% per decade) and the negative correlation between keratocyte density and stromal and total thickness (Jalbert et al., 2003).

The aim of the present study was to make a quantitative assessment of the age‐related changes occurring at the level of corneal innervations by means of in vivo confocal microscopy (IVCM), and to evaluate the changes visualized by optical microscopy and transmission electron microscopy (TEM). A thorough knowledge of the normal appearance of the corneal layers and their usual morphological variants is fundamental when applying IVCM to the anterior segment of the eye to identify corneal pathologies. MC (a noninvasive methodology) offers the advantage of allowing examination of the human cornea in its physiological state, avoiding the artifacts induced by an “ex vivo” study and permitting multiple examinations of the same cornea over time.

## MATERIALS AND METHODS

### Ethics Statement and Patients

The experimental protocol was approved by the local Ethics Committee and strictly adherent to the guidelines of the Declaration of Helsinki for research on human participants and in agreement with the ARVO declaration for use of human samples in ophthalmic and vision research. Informed consent was obtained from all subjects before they underwent any procedures.

A total of 20 patients underwent surgery to remove the wounded eye. Ten were young (mean age 28 years, three female and seven male) and 10 were elderly (mean age 76 years, five female and five male). The enucleation of the eye was performed following a traumatic eye injury. Another 40 patients (20 mean age <30 years and 20 mean age >70 years) were enrolled in our study for in vivo analysis using CM Laser IVCM.

Patients with traumatic injuries that did not involve the posterior and anterior segment of the eyeball were included in the study based on our inclusion criteria, while those with systemic or preexisting eye diseases were rejected in accordance with our exclusion criteria. Eligible subjects described themselves as “healthy.” Exclusion criteria included history and/or signs/symptoms of ocular surface disease as well as contact lens use. Further exclusion criteria included systemic conditions (including cardiovascular, metabolic, neoplastic, and psychiatric diseases) and pregnancy. In addition, all patients who had used any topical and/or systemic medications in the 3‐month period preceding the study were excluded. Subjects who underwent enucleation of the eyeball, following traumatic events, were included among the healthy subjects because they had not reported any damage to the corneal structures.

The samples were brought into our laboratory in 0.1 M Tris‐buffered saline with protease inhibitors, 0.025 M ethylene‐diamino‐tetraacetic acid (EDTA), 0.001 M benzamidine hydrochloride, 0.001 M phenylmethysulfonyl fluoride (PMSF), 0.01 M N‐ethylmaleimide [NEM] on wet ice.

Small pieces of the cornea, which included several nerve plexus filaments of the limbus and sub‐basal plexus, were dissected immediately after surgery (less than 2 min), prefixed with Bouin's fixative, and brought to our laboratories.

### Ethical Approval

“All procedures performed in studies involving human participants were in accordance with the Ethics Committee of Policlinico Umberto I and with the 1964 Helsinki declaration and its later amendments or comparable ethical standards.”

### Light Microscopy

After dissection, small pieces of the cornea were immersed in a phosphate buffer (PBS, 0.1 M, pH 7.4). They were then fixed with 10% buffered formalin, embedded in paraffin, and cut into serial sections (5 μm in thickness) using a rotative microtome. Sections were stained with hematoxylin and eosin (Mazzi, 1977) and then underwent morphological analysis.

### Transmission Electron Microscopy

Small samples of corneal tissue were fixed in 2.5% glutaraldehyde in 0.1 M PBS at pH 7.0 for 2 h, then washed three times in 0.1 M PBS (15 min × 3). Tissues were postfixed in osmium tetroxide (1%) for 1 h, then washed (15 min × 3) with distilled water and serially dehydrated in 30%, 50%, 70%, 96%, and 100% ethanol for 30 min in each case. Tissue fragments were placed in a 1/1 mixture of propylene oxide overnight, pure resin was then used for embedding and the samples were incubated at 60°C for 48 h. Ultrathin sections were obtained using a diamond knife (100 nm thick) on the Leica EM UC7 ultramicrotome (Leica, Germany) and picked up on the copper grids. The grids were then checked and photographed with TEM EM10 (Zeiss, Germany).

### In Vivo Confocal Microscopy

Forty patients underwent in vivo examination using CM Laser IVCM (Heidelberg Retina Tomograph 3 with the Rostock Cornea Module [HRT/RCM]; Heidelberg Engineering GmbH, Heidelberg, Germany). The images obtained were acquired approximately at the corneal apex bilaterally in all patients. Using the sequence mode, six to eight scans were performed on the full‐thickness of the central cornea. Applying this technique, the sub‐basal plexus is found in the sub‐epithelial area, immediately at or posteriorly to the basal epithelial layer and anteriorly to the Bowman layer, typically at a depth of 50–80 μm. The three most representative images of each epithelial layer and the sub‐basal nerve plexus of both eyes were prepared for analysis by a masked observer (P.V.). The noninvasiveness of the method makes it possible to examine the physiological and pathological state of the cornea with no time limits as well as to evaluate the average density of nerve fibers in the different groups. The degree of tortuosity, frequency of beadings, and length of the fibers were also assessed (Oliveira‐Soto and Efron, 2010; Rao et al., 2010). Data from the 20 patients were divided and analyzed for each age group. The patients were divided into two groups: patients under the age of 30 (n = 10) and patients over the age of 70. The mean age was 23.67 ± 4.28 years for the first group (n = 10) and 70.25 ± 6.81 years for the second group.

Morphometric quantification of the density of nerve fibers was obtained by photographs of stained sections using a Quantimet Leica 2000 image analyzer (Quantimet Leica Microsystems Imaging Solutions Ltd., Clifton Road Cambridge, CBI 3QH, U.K.). This instrument measures the following parameters: (1) number of nerve fibers counted in 10 fields randomly chosen by the observer, (2) percentage of the total area occupied by nerve fibers in the above 10 microscope fields, (3) number of observed varicosities, (4) number of crossings or intersections of the nerve fibers, (5) total perimeter of nervous structures. The software associated with the Quantimet Leica is able to count and express these areas in conventional units (C.U.), that is, as percentages of the area occupied by nerve fibers in relation to the total observed area.

Data obtained from different measurements of each photograph were averaged yielding a single value per patient. Means ± S.E.M. were then calculated and referred to each nerve fiber group (Serio, 1986).

### Variable Pressure Scanning Electron Microscopy

The Variable Pressure Scanning Electron Microscopy (VP‐SEM) instrument (Hitachi, SU3500; Japan) allows for the morphological analysis of the surface of hydrated biological specimens without preliminary chemical treatments or conductive coating and is also equipped with dual energy dispersive spectroscopy (dEDS) detectors mounted on opposite sides of the column. Each detector (Bruker XFlash® 6|60) has a large active area consisting of a 60 mm^2^ chip together with the slim‐line detector finger, which provides a large solid angle ideal for nano‐analysis (Matassa et al., 2016a; Matassa et al., 2016b). The XFlash® 6|60 is designed for use in applications with a relatively low X‐ray yield, delivering a good energy resolution for detecting a low energy range of atoms with a low atomic number. Using the dual EDS detector setting makes it possible to mitigate the shadowing effects. By adding the detected X‐ray photons or signal at the same time from the double detectors processing a large active area of detection, the dual EDS system allows faster EDS Spectra acquisition, reducing the time collection and avoiding any electron radian damage (or artifacts) on hydrated biological samples. The purpose‐built chemical identification apparatus is thus able to provide high performance simultaneous imaging and sensitive elemental analysis as well as the mapping of hydrated biological structures. Furthermore, hydrated bio‐samples were mounted on pure silicon wafers in order to avoid any fluoresce peak that might have influenced our EDS analysis.

## RESULTS

### Light Microscopy

**Figure 1:**
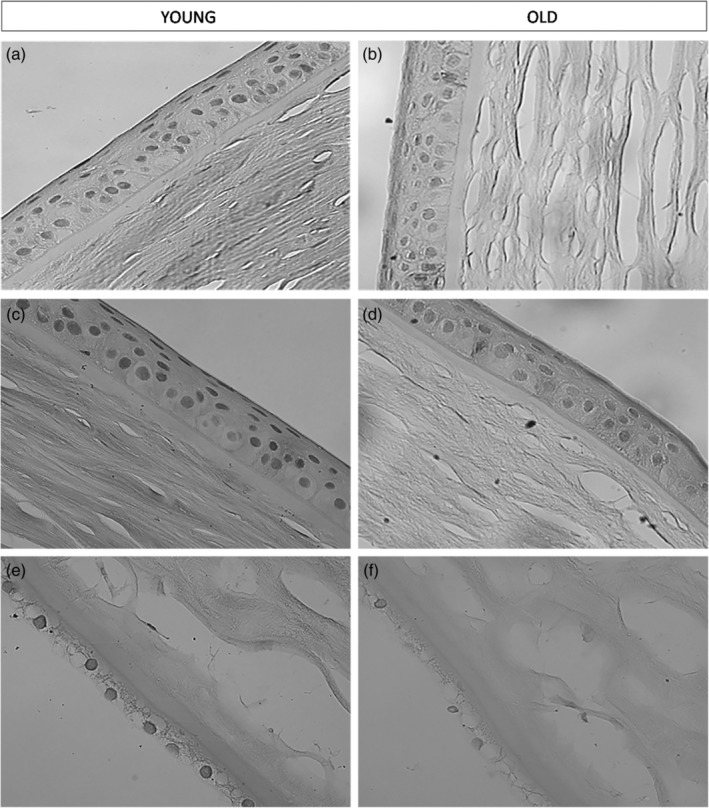
Age‐related changes in the corneal layers: Light microscopy. (a) and (c) Figures show the epithelial layer of the human cornea in a young subject. We can observe a contiguous and compact paving of normal epithelial cells (×40). (b) and (d) Figures show the epithelial layer of the human cornea in an elderly subject (×40). (e) Figure shows the endothelial layer of the human cornea in a young subject (×40). (f) Figure shows the endothelial layer of the human cornea in an elderly subject. We can observe that the endothelial cells are discontinuous and partially swollen (×40).

The results of our morphological study show that considerable changes take place in the structure of the cornea in older subjects compared to young ones. Relevant alterations in the cornea that occur with age include the thickening of both the epithelial and endothelial layers, the latter known as Descemet's membrane. Moreover, a reduction in the number of keratocytes was found (Fig. 1). In the group of elderly patients, the epithelial cells' borders are irregular, while in the group of younger patients, the borders appeared to be regular (Figs. 1a–1d). Figure 1e and 1f are micrographs of the endothelial layers of the human cornea in a young subject (Fig. 1e) and in an elderly subject (Fig. 1f). In Figure 1e, we observe a contiguous and compact paving of normal endothelial cells and the cells are separate and decomposed with hexagonal borders (young subject). In Figure 1a and 1c, a contiguous and compact layer of normal epithelial cells can also be seen. Figure 1f (elderly patient) shows corneal tissue from older subjects: the endothelial cells are discontinuous and partially swollen. A well‐documented loss of corneal endothelial cells is the most relevant and clinically appreciable age‐related change in the cornea.

### Transmission Electron Microscopy

We used TEM to study the corneal stroma. Corneal aging seems to be accompanied by structural and functional changes, which may be defined as clear modifications in important corneal features.

**Figure 2:**
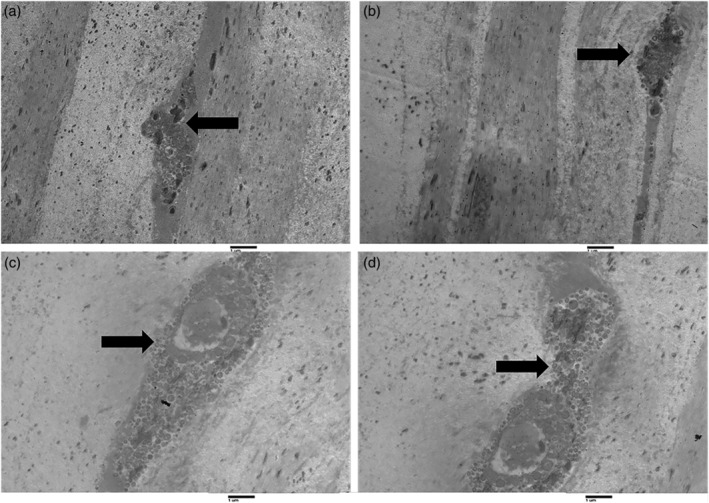
Transmission electron microscopy of longitudinal sections of the human cornea in young subjects. Figures (**a**) and (**b**) show two dendrites present in the corneal stroma of young subjects (arrows). Figures (**c**) and (**d**) show a different magnification of the same structure: dendrite with a big vesicle within the corneal stroma in a young subject (arrows; magnification ×2000).

**Figure 3:**
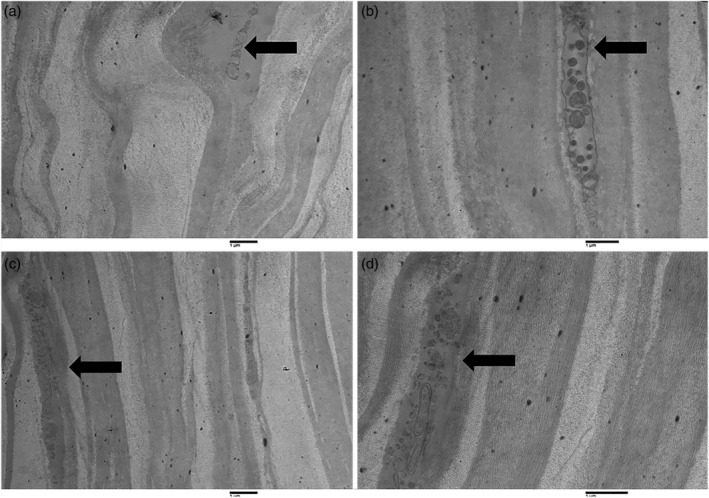
Transmission electron microscopy of longitudinal sections of the human cornea in elderly subjects. Figures (**a**) (magnification ×1,600) and (**b**) (magnification ×2,500) show two distal dendrites found in the corneal stroma of elderly subjects (arrows). Figures (**c**) (magnification ×1,600) and (**d**) (magnification ×3,150) show different magnifications of the same structure: nerve fiber, in particular, a non‐myelinated axon within the corneal stroma in an elderly subject (arrows).

Keratocyte density is reduced with age in the anterior and posterior stroma from the central or peripheral regions of the cornea. Corneal thickness was found to be significantly diminished in the group of elderly patients. These findings suggest that oxidative stress from mitochondria might produce corneal thinning with a concomitant reduced number of corneal stromal cells. However, fibers with a Type VII collagen appearance were found in situ at the epithelial basement membrane and, at intervals, individual or several collagen fibrils were described. Various bodies with collagen‐like cross striations were dispersed within the posterior portion of the basement membrane. Figures 2 and 3 are TEM micrographs of the corneal stroma in a young (Fig. 2) and in an elderly (Fig. 3) patient—stromal nerve fibers are evident. Keratocytes in elderly patients were irregular and reduced in number. Figures 2b and 3a are TEM micrographs of the sub‐basal plexus in the human cornea in a young (Fig. 2b) and in an elderly (Fig. 3a) patient. In the older subject, the nerve fibers are markedly reduced. Figure 2c is a TEM micrograph of a thin nervous filament harvested near the corneal limbus from a young subject in which numerous thick and thin nerve fibers may be seen. Figure 3c is a TEM micrograph of a thin nervous filament from an elderly patient, showing a marked reduction in the thin nervous fibers while the thick ones were well preserved. Moreover, in images obtained with TEM, we observed a variation in the stromal collagen fibers, which showed a statistically significant thickening in older subjects. We also conducted quantitative and statistical analyses of the experimental results. The results of these analyses are reported in Table 1, which shows the quantification of collagen fibers in the stromal layer of the human cornea in young and elderly subjects.

**Table 1:** Quantitative Analysis of Collagen Fibers on Images Obtained with TEM

Numbers of collagen fibers in corneal fascicles	Younger subjects (n = 10)	Older subjects (n = 10)	*P* a
Total collagen fibrils in each fibers^b^	28.3 ± 1.2	15.4 ± 1.4	<0.0001
Thin collagen fibers %^c^	61.5 ± 3.1	4.3 ± 1.8	<0.0001
Thick collagen fibers %^c^	37.3 ± 4.6	91.5 ± 3.1	<0.0001
Swelled collagen fibers %^c^	1.4 ± 0.6	31.6 ± 4.4	<0.0001
Total number of corneal collagen fibers^b^	36.4 ± 5.3	35.3 ± 1.2	=0.5302

a *P* is of high significance if >0.001.

b Results are expressed as C.U. ± SEM.

c Results are expressed as %.

### In Vivo Confocal Microscopy

**Figure 4:**
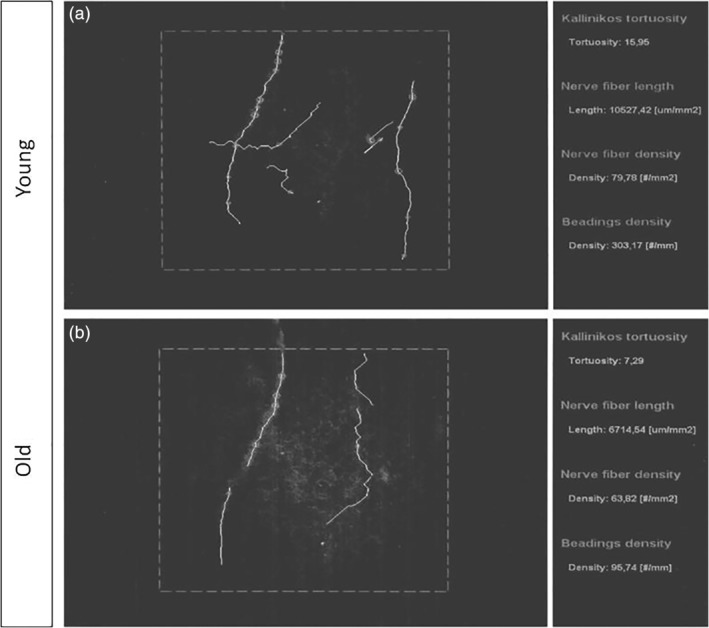
Sub‐basal nerve plexus of human corneas: in vivo confocal microscopy. (**a**) Sub‐basal nerve plexus of young subjects. (**b**) Sub‐basal nerve plexus of elderly subjects.

Our study is not observational and thus a quantitative analysis was performed. To evaluate age‐related changes in the various corneal layers, we performed a quantitative in vivo corneal analysis by CM (Fig. 4). Our clinical results are reported in Tables 2, 3, 4. Table 2 shows the quantification of nerve fibers in the sub‐basal plexus of the human cornea in young and elderly subjects. The results are expressed in Conventional Units (CU) ± SEM. *P* is significant when <0.001. The number of nerve fibers per mm^2^ of corneal sub‐basal layer was 16.7 ± 3.2 in young subjects, whereas in elderly subjects these values were 6.6 ± 1.7 (*P* < 0.001). Therefore, a strong decrease in nerve fibers can be observed in older subjects. The total area occupied by nerve fibers, expressed as a percentage of the total area observed, was 31.4 ± 2.8 in young subjects and 18.5 ± 1.9 in the elderly ones. Moreover, nervous varicosities (9.6 ± 3.7), nerve crossings (18.3 ± 4.1), and perimeter of total nerve fibers (41.6 ± 5.7) were found in young subjects (Table 2), whereas these values were markedly reduced in elderly subjects (3.4 ± 1.1), (7.9 ± 2.6), (26.5 ± 3.6), respectively. Table 3 shows quantitative and statistical results for both epithelial and endothelial cells of the human cornea in young and elderly patients. CM analysis of the cornea of 40 living patients showed an average density of nerve fibers equal to 94 ± 13.2 in subjects under the age of 30. In the group consisting of patients over the age of 70, the density of nerve fibers was 82.3 ± 10.05. The average number/frequency of beadings was 532.33 ± 159.42 in young patients and 413.02 ± 90.35 in subjects over the age of 70 (Table 4).

**Table 2:** Data of Confocal Microscopy in Living Subjects. Quantification of Nerve Fibers in Sub‐Basal Plexus of the Human cornea

Nervous structures	Younger subjects (n = 10)	Older subjects (n = 10)	*P* b
Number of nerve fibers × mm^2^ a	16.7 ± 3.2	6.6 ± 1.7	<0.0001
Percentage of total area occupied by nerve fibers^a^	31.4 ± 2.8	18.5 ± 1.9	<0.0001
Nervous varicosities^a^	9.6 ± 3.7	3.4 ± 1.1	<0.0001
Nerve fibers crossings^a^	18.3 ± 4.1	7.9 ± 2.6	<0.0001
Total perimeters of nerve fibers^a^	41.6 ± 5.2	26.5 ± 3.6	<0.0001

a The results are expressed in C.U. ± SEM in every optical field.

b *P* is of high significance if <0.001.

**Table 3:** Data of Confocal Microscopy in Living Subjects. Quantification of Epithelial and Endothelial Cells in the Human Cornea

Corneal epithelial and endothelial cells	Younger subjects (n = 10)	Older subjects (n = 10)	*P* a
Epithelial cells hexagonal × mm^2^ b	98.1 ± 0.9	12. 3 ± 3.1	<0.0001
Endothelial cells well preserved %^c^	99.5 ± 0.4	36.4 ± 3.1	<0.0001
Endothelial cells swelled %^c^	1.2 ± 0.9	37.4 ± 4.5	<0.0001
Endothelial cells destroyed %^c^	0.3 ± 0.2	21.3 ± 3.7	<0.0001

a *P* is of high significance if <0.001.

b Results are expressed as C.U. ± SEM.

c Results are expressed as percentage of cells in the 10 optical fields ± SEM.

**Table 4:** Data of Confocal Microscopy in Living Subjects

	Young subjects (n = 20)	Old subjects (n = 20)	*P* a
Density of nerve fibers	94 ± 13.2^b^	82.3 ± 10.05^b^	=0.0373
Beadings	532.33 ± 159.42^c^	413.02 ± 90.35^c^	=0.0543

a *P* was calculated by confront of the values of younger and older subjects.

b Results expressed as μm/mm^2^ of corneal tissue.

c Results are expressed as numbers of beadings in 1 mm^2^ of corneal tissue.

### Variable Pressure Scanning Electron Microscopy (VP‐SEM)

**Figure 5:**
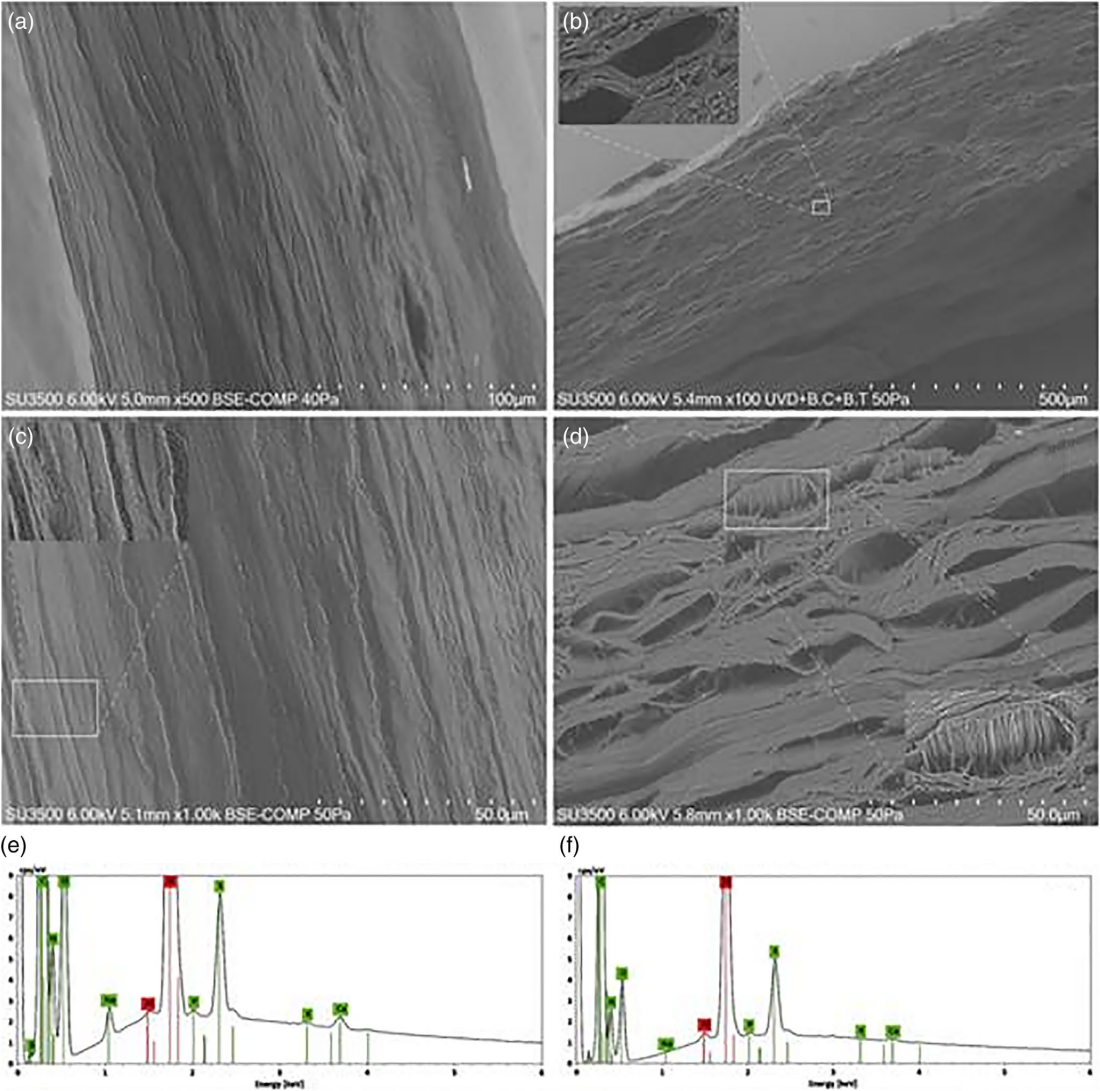
Morphometric observations and chemical analyses of hydrated human corneas of young and elderly patients: (**a**) cross section of young and (**b**) of elderly subjects. Inset magnified with line rectangle. (**c**) Stromal cross sectioning of young and (**d**) old cornea. (**e**) and (**f**) EDS spectra probed on micrometric area of Figures c and d (elements identified: C, N, O, Na P, S, K, and Ca with green background, while the remaining elements in red belong to the background of the VP‐SEM camera). [Color figure can be viewed at http://wileyonlinelibrary.com]

Surface morphological observations of the cornea samples were made and their chemical composition was determined. A cross‐sectional view of young corneas shows the presence of long aligned collagen lamellae along the stromal region (Fig. 5a). On the other hand, a similar section of an old cornea shows a rough, undulating surface with adjacent curvilinear stromal lamellae, each approximately 0.65 μm thick (Fig. 5b).

By slightly increasing the magnifications, we were able to observe the extracellular matrix, essential for regulating the cohesion processes, including the aligned collagen lamellae of the stromal region (Fig. 5c). The inset shows an in vivo human corneal section. Old in vivo human corneas clearly exhibit the presence of different sized micro‐cavities, as shown in Figure 5d. The presence of such cavities is probably due to deficiencies in the stromal extracellular matrix essential for the preservation of the few aligned collagen lamellae (Fig. 5d). The corresponding inset shows a natural loss of cohesiveness within a single lamella, in which it is possible to observe parallel collagen bundles, about 0.41 μm in size. The absence of the extracellular matrix is critical not only for regulating the self‐assembling cohesiveness of the collagen lamellae, but also for assessing the photomechanical properties of the cornea. Hence, we also performed EDS analyses of micrometric areas in both young and old corneas in order to understand the physical–chemical variations that take place during the transition of the cornea from young to old age. Figure 5e shows the EDS spectrum obtained from the sample of Figure 5c in a young subject. The interesting elements are highlighted in green and show high counts of C, N, O, and S chemical species, while lower peaks belong to the Na, P, K, and Ca species, and the red one to the background of the instrument (Al and Si elements of the holder sample). In short, the main differences in the X‐ray intensity peaks between the young and old subjects can be observed in the O, Na, S, K, and Ca peaks. Among the most prominent macromolecules of the extracellular matrix, the corneal sulfate proteoglycans, composed of protein covalently linked with the glycosaminoglycan chain, play a fundamental role in mechanically assembling the collagen fibers and their subsequent development (Michelacci, 2003). These corneal macromolecules are literally made up of carbon, oxygen, and sulfur species. Therefore, the loss of intensity of the corresponding X‐ray peaks in the old cornea shown in Figure 5f corresponds to a loss of extracellular matrix, corroborated by the presence of cavities among the collagen lamellae in Figure 5d. It is interesting to observe the physiochemical alteration of the amount of Ca in older subjects. Indeed, we can observe a loss of about of 50% compared to the calcium X‐ray peaks in a young cornea.

## DISCUSSION

In this study, the full‐thickness of the corneal structure was analyzed. The epithelial layer and the endothelial layer of the cornea were analyzed by optic microscopy. Moreover, age‐related changes in the density of several corneal cells, such as basal epithelial cells, keratocytes, and endothelial cells, were analyzed thoroughly. Using optic microscopy, we could observe a decrease in ECD with age and associated with an increase in endothelial cell size.

Although different nerve fibers were shown in all corneal layers, the pattern of distribution of catecholaminergic fibers after the occurrence of a superficial lesion to the cornea suggests that the superficial and deep nerve fibers may have a different distribution, as is reportedly the case in other organs as well (Cavallotti et al., 1999). The epithelium of the cornea acts as a barrier against environmental agents and plays a crucial role in the movement of water and ions through the cornea; however, this function appears to deteriorate with age, resulting in a breakdown of the epithelial barrier function. A common effect of age on the human cornea is a progressive decrease in ECD. However, measurement of this feature is not a reliable index of the age of the cornea because there is a large range of ECD within normal populations (Laing et al., 1976; Roszkowska et al., 2004). Corneal images acquired by in‐vivo specular and CM provide clinical information on changes in corneal structures that occur during aging. Indeed, the normal hexagonal shape of endothelial cells is usually affected by age, with older patients showing discontinuous and partially swollen endothelial cells (Gambato et al., 2015). Moreover, corneal ECD decreases as well as the number of keratocytes.

Without a proliferative response to cell loss, the endothelial covering in the posterior part of the cornea is insured by a gradual enhancement in size of the residual cells, which results in an increased cellular pleomorphism and a reduction in the percentage of hexagonal cells during aging. The analysis of endothelial cells provides important clinical information regarding corneal function and viability. Numerous studies have been published regarding the age‐related decrease in ECD and changes in their morphology. These studies show that cellular polymegathism and cellular pleomorphism increase with age. Indeed, Bourne and Kaufman (1976) reported a 0.39%/year reduction in central endothelial cells with age, while Hollingsworth et al. (2001) reported that ECD is reduced at a rate of 0.33%/year.

IVCM demonstrated that the human sub‐basal nerve network is oriented vertically at the central corneal apex. Our study demonstrated a significant difference between the young and elderly subjects, thus confirming the results obtained by Grupcheva et al. (2002), who carried out a quantitative evaluation of the nerve plexus of human corneas by means of IVCM and analysis software. In the present study, analytic software allowed us to identify the individual axons present in a bundle of nerve fibers; thus, making it possible to identify the individual age‐related axon degeneration and to properly evaluate the decrease in nerve density in the sub‐epithelial and corneal plexi. Central corneal sensitivity appears to remain unchanged until middle age, whereas a significant decrease is observed in patients over the age of 70. Corneal sensitivity decreases significantly throughout the human life span. Results reveal an initial decrease in corneal sensitivity in the peripheral area, followed by a decrease in the entire cornea. It is important to be able to define normal corneal appearance together with the effects of aging on corneal layers before attempting to identify pathological changes (Stepp et al., 2017). Our experimental study was based on a quantitative and morphological analysis of age‐related changes in the corneal structures in physiological conditions (Cocco et al., 1999). The decreased ECD may be due to a global reduction in corneal nerves during the aging process. The concomitant reduction in nerves and the endothelium may be related to corneal nerves and their possible role in the homeostasis of endothelial functions. On the other hand, both nerves and endothelial cells may be decreased as a result of a pathogenic mechanism such as inflammation. Inflammatory processes associated with aging may modify corneal homeostasis, producing not only nerve injury, but also dysfunction and/or structural alteration of the endothelial layer. The corneal nerve network has an important effect on corneal trophism and contributes to the preservation of a healthy corneal surface. Changes in corneal innervation determine corneal alterations such as neurotrophic keratitis. The demonstration of the existence of nerve bundle alterations in old patients points to the possible usefulness of neuroprotective and/or neurotrophic ocular drops in such patients. Our experimental study is the first of its kind to describe the presence of such alterations as a function of the aging process. Nevertheless, we are unable to determine whether the changes in corneal innervation are primary or secondary to the epithelial alterations.

Moreover, our study provides information regarding differences in X‐ray intensity peaks for O, Na, S, K, and Ca between young and elderly subjects. This chemical behavior may be related to the degradation of the proteins containing calcium ions binding oxygen sites since the O X‐ray peaks also decrease in old corneas. The potassium ions display the same physical–chemical behavior, which depends on the degree of sulfonation of the sulfated glycosaminoglycans responsible for the maturation of the corneal extracellular matrix. This mechanism would also explain the increased thickness of the collagen fibers present in the corneal stroma of older subjects (Koudouna et al., 2012). Finally, the loss of sodium observed in the stroma of old corneas may be related to the loss of the osmotic forces, which are critical to preserving the transparency of adult corneas. In our study, we have demonstrated that although multiple factors may contribute to physiological corneal aging, the decrease in the number of nerve fibers associated with the loss of corneal sensitivity plays a central role in the whole degenerative process, including the functionality of endothelial and epithelial cells. Moreover, these observed changes may have important consequences. The demonstrated presence of such alterations in corneal innervation during aging might lead to the development of new drugs potentially useful in the prevention of several eye diseases that develop during the physiologic aging process.

## CONCLUSIONS

The aim of our study was to determine whether corneal aging may be associated with an alteration of corneal innervation.

Deepening our knowledge of the effects of aging on corneal morphology and functionality can help us to understand whether degenerative disorders are caused by physiological aging or specific pathologies.

The reason for the gradual loss of both epithelial and corneal cells with age remains unclear. Green (1995) suggested that during eye aging, the process occurs as an epiphenomenon of the deterioration of enzymes that metabolize and detoxify hydrogen‐peroxidase, thus inducing irreversible negative effects on various ocular tissues. Such effects may lead to cataract formation in the lens as well as a reduction in corneal endothelial cells. Our results support the theory of an age‐related reduction in nerve fibers, thus explaining the decreased corneal sensitivity and related dysfunctions that have been observed in the elderly.

## CONFLICT OF INTEREST

The author(s) declare no potential conflicts of interest regarding the research, authorship, and/or publication of this article.
